# Hormone actions controlling sex-specific life-extension

**DOI:** 10.18632/aging.101396

**Published:** 2018-03-03

**Authors:** Michael Garratt, Michael B. Stout

**Affiliations:** 1 Department of Pathology, University of Michigan Medical School, Ann Arbor, MI 48109, USA; 2 Department of Nutritional Sciences, University of Oklahoma Health Sciences Center, Oklahoma City, OK 73104, USA; 3 Reynolds Oklahoma Center on Aging, University of Oklahoma Health Sciences Center, Oklahoma City, OK 73104, USA; 4 Harold Hamm Diabetes Center, University of Oklahoma Health Sciences Center, Oklahoma City, OK 73104, USA

**Keywords:** sex, 17α estradiol, acarbose, metabolism, androgen, estrogen, aging

In most sexually reproducing species, including humans, males and females have different lifespans [1]. The sexes also frequently differ in their age-associated functional decline and disease incidence, highlighting a general disparity in the way males and females age. Understanding the causes for these sex-differences in aging could lead to ways of ameliorating age-related human disease in one or both sexes.

It is now becoming recognized that the responsiveness to interventions that extend lifespan in model organisms can differ between males and females [2]. Two of the strongest examples of sex-specific life-extension have been observed in mice treated with acarbose – an anti-diabetes drug that slows carbohydrate digestion - and 17α- estradiol (17α-E2) - a ‘nonfeminizing’ steroid with reduced affinity for the classical estrogen receptors [3]. In spite of their predicted disparate modes of action, both treatments extend median lifespan in males by nearly 20%, yet have much smaller (acarbose) or undetectable (17α-E2) effects in females. Understanding the potential pathways through which these compounds extend male lifespan, and the underlying causes for the observed sex-specificity, could provide important insight into the causes of sex-differences in aging in mammals.

Gonadally-derived hormones control many of the sex-differences observed in mammalian biology and previous reports have established that endogenous androgens and estrogens can influence the lifespan (Reviewed in [1]). In a recent study Garratt et al. [4] sought to determine if endogenous gonadal hormones were central to sex-specific responses to acarbose and 17α-E2. The authors first demonstrated that acarbose and 17α-E2 improved glucose tolerance and enhanced hepatic mTORC2 signaling in male mice, with smaller effects on these parameters in females. These findings highlight the involvement of glucose metabolism and insulin signaling as important factors for survival in male mice. Moreover, these male-specific responses were inhibited if the animals had been castrated prior to treatment initiation (at 3 months of age), suggesting male-specific responsiveness was dependent upon circulating testis-derived hormones. The authors also evaluated intervention-responsiveness in females that had been ovariectomized. With acarbose treatment, ovariectomized females demonstrated enhanced hepatic mTORC2 signaling, which was not observed in sham-operated females. This suggests that one reason females show less of a response to acarbose is because they are exposed to circulating ovarian hormones.

This report by Garratt et al. strongly suggests that some physiological and biochemical sex-specific responses to acarbose and 17α-E2 are associated with gonadal hormone production. If sex-specific longevity responses to these treatments are also linked to production of gonadal hormones, consideration must to given to how these hormones modulate downstream signaling pathways when attempting to translate these and related treatments into the clinical setting. Understanding the underlying hormonal causes of these sex-specific treatment responses and the pathways through which they operate may also provide a general insight into the causes for sex-specific aging. One simplistic argument for why castration would inhibit treatment responses to acarbose and 17α-E2 is that male gonads produce a stimulus (e.g. testosterone) that shortens male lifespan relative to females, and that acarbose and 17α-E2 ameliorate these effects. Previous studies have shown that male castration does indeed extend lifespan, including in rodents, indicating that the presence of male gonads provides a constraint on male lifespan [1]. However, there is one key observation that suggests such a model is too simplistic: male mice treated with 17α-E2 live slightly but significantly longer than both control and 17α-E2-treated female mice of the same strain studied in parallel [3]. Thus, 17α-E2 does not simply protect against a male-specific cause of ageing that leads males to live shorter than females, since this treatment can push male lifespan even higher than that of females.

Another recent report by Steyn et al. [5] described how the acute effects of 17α-E2 on male mouse feeding behavior, body mass, and adiposity are dependent upon functional hypothalamic pro-opiomelanocortin (Pomc) expressing neurons. These neurons modulate energy balance by stimulating satiety in response to activation by circulating factors, including leptin, insulin, and 17β-estradiol [6]. While this new study and the aforementioned work on castration examined the effects of 17α-E2 on metabolic parameters over different time frames, both reports provide insight into how responsiveness to 17α-E2, and perhaps other sexually-dimorphic lifespan extending interventions, may be linked to both testosterone activity and hypothalamic control of metabolism. Interestingly, castration has been reported to substantially reduce the *Pomc* mRNA expression of in the hypothalamus, which is at least partially dependent on aromatization of testosterone to estrogen [7]. This observation provides one potential way that the studies by Garratt et al. and Steyn et al. may be linked. A mechanism of action whereby 17α-E2 alters testosterone metabolism either in the brain or peripheral tissues, as reported *in vitro* [8], or is itself signaling and/or metabolized in a sex-specific manner, could explain its sexually dimorphic effects on survival. Understanding these mechanisms, particularly the causal requirement of steroid activity in particular tissues including the brain, could lead to the development of more specific androgen and/or estrogen pathway modulators that help to slow aging in one or both sexes while minimizing adverse health outcomes.
